# Sunflower WRINKLED1 Plays a Key Role in Transcriptional Regulation of Oil Biosynthesis

**DOI:** 10.3390/ijms23063054

**Published:** 2022-03-11

**Authors:** Audrey R. Q. Lim, Que Kong, Sanjay K. Singh, Liang Guo, Ling Yuan, Wei Ma

**Affiliations:** 1 School of Biological Sciences, Nanyang Technological University, Singapore 637551, Singapore; limr0029@e.ntu.edu.sg (A.R.Q.L.); quekong@ntu.edu.sg (Q.K.); 2 Department of Plant and Soil Sciences, Kentucky Tobacco Research and Development Center, University of Kentucky, Lexington, KY 40546, USA; sanjaysingh@uky.edu (S.K.S.); lyuan3@uky.edu (L.Y.); 3 National Key Laboratory of Crop Genetic Improvement, Huazhong Agricultural University, Wuhan 430070, China; guoliang@mail.hzau.edu.cn; 4 Hubei Hongshan Laboratory, Wuhan 430070, China

**Keywords:** gene expression, plant oil biosynthesis, sunflower, transcription factor, transactivation, WRI1

## Abstract

Sunflower (*Helianthus annuus*) is one of the most important oilseed crops worldwide. However, the transcriptional regulation underlying oil accumulation in sunflower is not fully understood. WRINKLED1 (WRI1) is an essential transcription factor governing oil accumulation in plant cells. Here, we identify and characterize a sunflower ortholog of *WRI1* (*HaWRI1*), which is highly expressed in developing seeds. Transient production of HaWRI1 stimulated substantial oil accumulation in *Nicotiana benthamiana* leaves. Dual-luciferase reporter assay, electrophoretic mobility shift assay, fatty acid quantification, and gene expression analysis demonstrate that HaWRI1 acts as a pivotal transcription factor controlling the expression of genes involved in late glycolysis and fatty acid biosynthesis. HaWRI1 directly binds to the *cis*-element, AW-box, in the promoter of *biotin carboxyl carrier protein isoform 2* (*BCCP2*). In addition, we characterize an 80 amino-acid C-terminal domain of HaWRI1 that is crucial for transactivation. Moreover, seed-specific overexpression of *HaWRI1* in Arabidopsis plants leads to enhanced seed oil content as well as upregulation of the genes involved in fatty acid biosynthesis. Taken together, our work demonstrates that HaWRI1 plays a pivotal role in the transcriptional control of seed oil accumulation, providing a potential target for bioengineering sunflower oil yield improvement.

## 1. Introduction

Plant oils (also known as vegetable oils) are mainly biosynthesized and accumulated in seeds as triacylglycerol (TAG) to support seed germination and seedling establishment as a main carbon and energy resource. Vegetable oils are important components of the human diet and essential as a renewable source for biofuels and raw materials for industrial applications [1,2,3,4,5]. Sunflower (*Helianthus annuus*) was cultivated in North America thousands of years ago and is currently one of the most prominent global oilseed crops. Sunflower seeds are a central source of edible oil and an important food ingredient. Sunflower seeds comprise approximately 35–42% of oil that is enriched in linoleic acid (55–70%) [6]. The seed oil accumulation significantly increases between 12 and 28 days after flowering (DAF) [7,8].

A wealth of information on TAG biosynthetic pathways and the associated molecular mechanisms has become available in recent years [2,9,10,11,12,13]. *WRINKLED1* (*WRI1*) encodes an APETALA2 (AP2) transcription factor [14,15] and fosters the conversion of sucrose to oils via the activation of genes encoding enzymes that are involved in late glycolysis and fatty acid synthesis [16,17]. The Arabidopsis *WRI1* loss-of-function mutant (*wri1-1*) exhibits an approximate 80% decrease of seed oil content [18]. Comparative transcriptomic analysis shows that the majority of the downregulated genes in *wri1-1* encode fatty acid biosynthetic and glycolysis enzymes [19], and these genes are direct targets of AtWRI1 [16,17]. The AtWRI1 binding sites in the promoters of the target genes comprise a consensus sequence [CnTnG](n)7[CG] (designated as the AW-box), where n represents any nucleotide [17]. *WRI1* orthologs have been recently discovered from diverse monocot and dicot plant species, such as *Avena sativa* [20,21], *Brachypodium distachyon* [22], *Brassica napus* [23], *Camelina sativa* [24], *Elaeis guineensis* [25,26], *Glycine max* [27], *Jatropha curcas* [28], *Oryza sativa* [29], and *Zea mays* [30,31]. Ectopic expression of *AtWRI1* and *WRI1* orthologs generates enhanced oil in seeds and vegetative tissues [14,22,23,27,31,32]. The function of AtWRI1 is mediated at the posttranslational level and affected by the 26S proteasome-mediated degradation [33]. Structurally, AtWRI1 contains three intrinsically disordered regions (IDRs), a C-terminal transactivation domain (TAD), and a PEST motif that mediates protein degradation [34]. The protein–protein interaction network of AtWRI1 consists of ever-expanding partners, such as BTB/POZMATH (BPM; CULLIN3 (CUL3)-based E3 ligase adaptor) [33], 14-3-3 s [35], SNF1-related protein kinase KIN10 [36,37], and TCP4 transcription factor [38]. The large number of interacting partners authenticate the central roles of WRI1 and enable the fine-tuning of the spatiotemporal regulation of plant oil biosynthesis.

Despite the scientific and economic values, literature on the molecular mechanisms of the transcriptional regulation of sunflower lipid biosynthetic pathways remains insufficient. In this study, we characterized the sunflower *WRI1* ortholog (*HaWRI1*) for its roles in regulating plant oil accumulation. We conducted detailed molecular and biochemical analyses to validate the functions of HaWRI1 in various aspects, such as stimulation of TAG biosynthesis, transactivation of the promoters of genes involved in fatty acid biosynthesis, and binding properties to AW-box. We also generated stable transgenic Arabidopsis overexpressing *HaWRI1* and demonstrated that *HaWRI1* overexpression effectively boosts seed oil production. The evidence we provided in this study not only elucidated the molecular function of HaWRI1 from the perspective of fundamental research but also highlighted the translational potential of HaWRI1 for bioengineering oil yield improvement in major oilseed crops.

## 2. Results

### 2.1. Identification of WRI1 Ortholog in H. annuus

**Figure 1:**
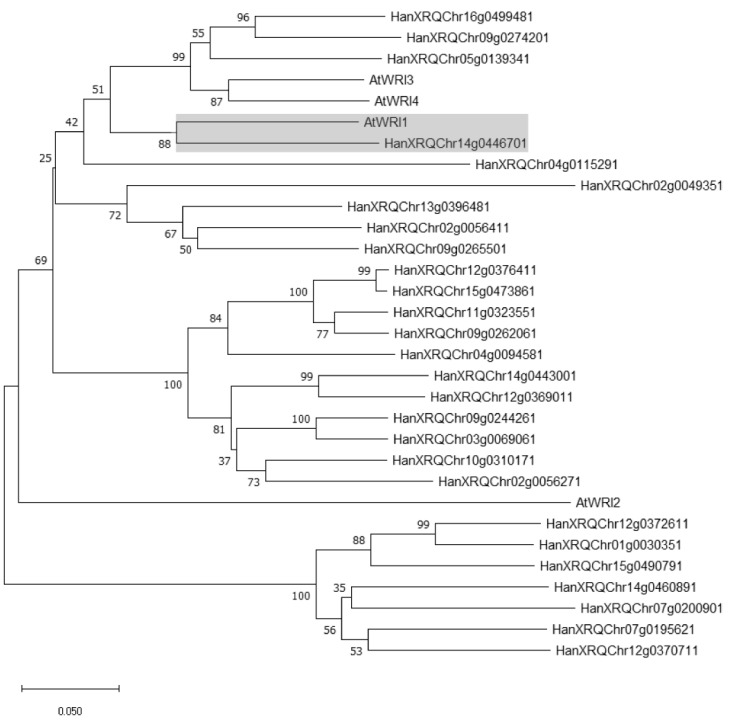
Phylogenetic analysis of AtWRI1 homologs from *H. annuus*. The phylogenetic tree was created using protein sequences of AtWRI1 and all double AP2 domain-containing proteins. The tree was constructed by MEGA X using the neighbor-joining method. The number of bootstrap replicates is 1000. Putative WRI1 ortholog identified from *H. annuus* was highlighted by a gray box.

We conducted a BlastP analysis using Phytozome (https://phytozome-next.jgi.doe.gov/, last accessed on 15 January 2022) to identify putative AtWRI1 orthologs in the *H. annuus* protein database. Our initial analysis led to the identification of 27 double AP2-domain-containing proteins. We next performed phylogenetic analysis using MEGA X, which revealed that one protein (HanXRQChr14g0446701) was the closest ortholog to AtWRI1 (Figure 1). HanXRQChr14g0446701 was thus termed HaWRI1. Further analysis revealed that AtWRI1 and HaWRI1 shared 52% amino acid sequence identity (Supplementary Figure S1). The “VYL” motif in the AP2 domain vital for the function of AtWRI1 [26] contains a conservative substitution in HaWRI1 as “IYL” (Supplementary Figure S1). The protein disorder prediction algorithm revealed that HaWRI1 had a disorder value of 55% (Supplementary Figure S2A), comparable to that of AtWRI1 [34]. Further in silico analysis revealed three IDRs in HaWRI1 (Supplementary Figure S2B), organizationally similar to AtWRI1 [34].

We next conducted a phylogenetic analysis of HaWRI1 with WRI1s identified from other plants and showed that HaWRI1 was grouped in the same clade with AtWRI1 and WRI1s from dicots, such as soybean, *Brassica napus*, and camelina (Supplementary Figure S3).

### 2.2. HaWRI1 Is Highly Expressed in Developing Seeds

**Figure 2:**
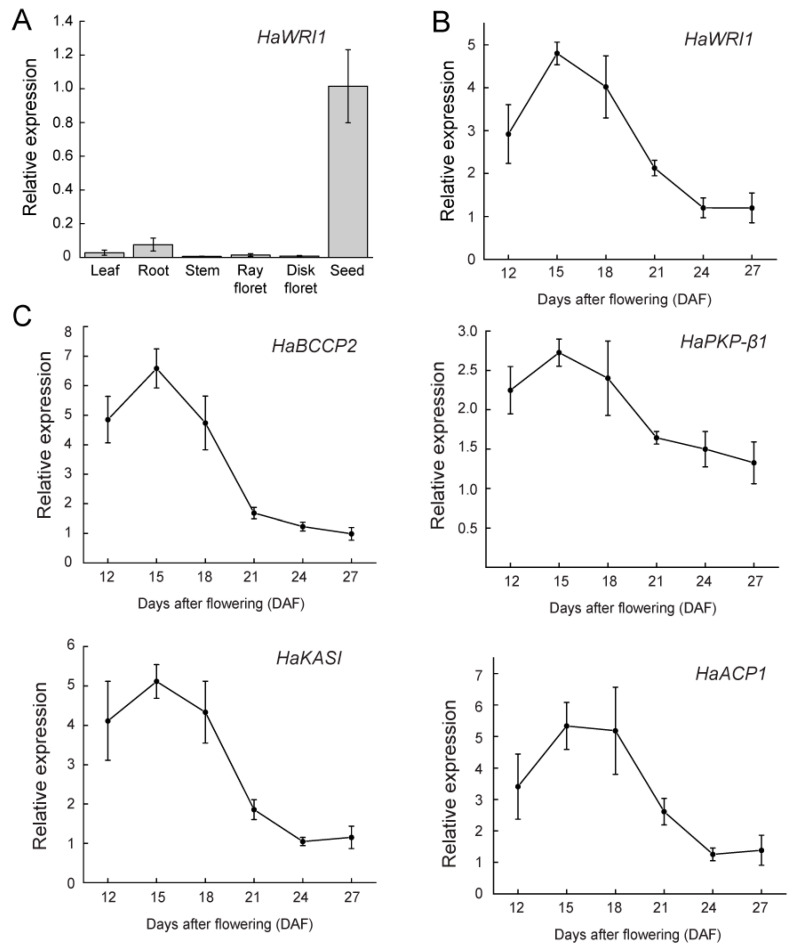
Expression analysis of *HaWRI1* and WRI1 target genes in a variety of *H. annuus* tissues by quantitative real-time PCR (qRT-PCR). (**A**) *HaWRI1* expression in different tissues of *H. annuus* plants as indicated. Results are shown as means ± SE (*n* = 3). (**B**) *HaWRI1* expression at various stages during seed development [12–27 days after flowering (DAF) as indicated]. Results are shown as means ± SE (*n* = 4–5). (**C**) Expression analysis of selected genes known as WRI1 targets in developing seeds of *H. annuus*. Expression level of *HaBCCP2, HaPKP-β1, HaKASI,* and *HaACP1,* at various stages during seed development (12–27 DAF as indicated) was quantified by qRT-PCR. Results are shown as means ± SE (*n* = 4–5).

To determine the spatial expression of *HaWRI1*, we used the available RNA-seq data from pistil, stamen, leaf, root, and seed to perform coexpression analysis. As shown in the heatmap, similar to many plant oil biosynthetic pathway genes, such as *biotin carboxyl carrier protein isoform 2* (*BCCP2*), *acyl carrier protein 1* (*ACP1*)*, plastidic pyruvate kinase beta subunit 1* (*PKP-β1*)*, 3-ketoacyl-acyl carrier protein synthase I* (*KASI*)*,* and *enoyl-ACP reductase 1* (*ENR1*), *HaWRI1* is highly expressed in seeds (Supplementary Figure S4). Additionally, expression of the B3-domain transcription factor FUSCA3 (*FUS3*), a known regulator of oil biosynthesis [39], is significantly higher in seeds. To validate the RNA-seq data, we examined the expression levels of *HaWRI1* in various tissues of sunflower using qRT-PCR. Similar to the RNA-seq data, *HaWRI1* displayed the highest expression in developing seeds with moderate expression in roots (Figure 2A). The preferential expression of *HaWRI1* in developing seeds is similar to those of *AtWRI1*, *GmWRI1s*, and *CsWRI1s* [14,24,27]. To further gain insights into *HaWRI1* abundance during seed development, we conducted a detailed time-course expression analysis using qRT-PCR. *HaWRI1* exhibited the highest level of expression on 15 DAF and was gradually reduced upon seed maturation (Figure 2B). Furthermore, we found that the temporal expression patterns of several *WRI1* targets (known to be involved in fatty acid biosynthesis) were similar to that of *HaWRI1* (Figure 2C). Together, our results suggest that *HaWRI1* is preferentially expressed in seeds and strongly upregulated during seed development and might play an essential role in mediating seed oil accumulation.

### 2.3. Subcellular Localization of HaWRI1

To visualize the HaWRI1 subcellular localization, we transiently produced a N-terminal YFP fusion of HaWRI1 (YFP-HaWRI1) in *N. benthamiana* leaves. As shown in Supplementary Figure S5, the fluorescence signal of YFP-HaWRI1 was detected in the nucleus, suggesting that HaWRI1 was nucleus-localized.

### 2.4. Transient Production of HaWRI1 Stimulates TAG Biosynthesis in N. benthamiana Leaves

**Figure 3:**
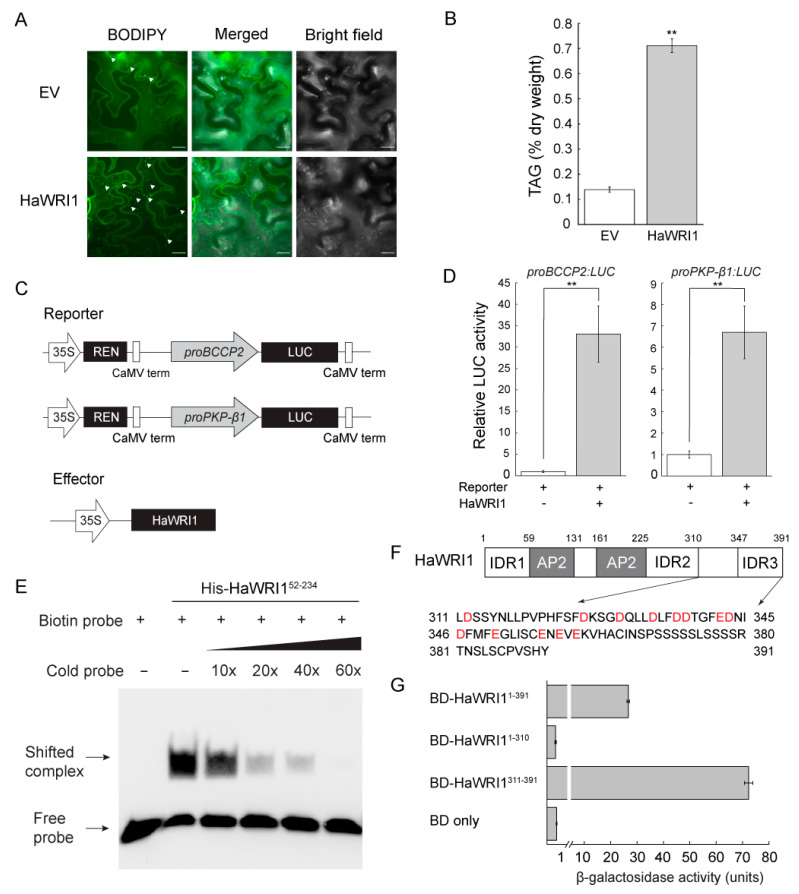
Functional analysis of HaWRI1. (**A**) Oil droplets in *N. benthamiana* leaves transiently expressing *HaWRI1*. Empty vector (EV) is used as a control. Confocal fluorescence images of *N. benthamiana* leaf mesophyll tissues displaying oil droplets stained with BODIPY (as indicated by the white arrows) were shown. Scale bar is 20 μM. (**B**) TAG content in *N. benthamiana* leaves transiently producing HaWRI1. Results are shown as means ± SE (*n* = 4). “**” indicates a significant difference (*p* < 0.01, Student’s *t*-test) between HaWRI1 and EV. (**C**) Schematic diagram of constructs used in the dual luciferase assay in *N. benthamiana* leaves through transient expression. (**D**) Transactivation activity of HaWRI1 on the promoters of *BCCP2* and *PKP-β1* (*proBCCP2* and *proPKP-β1*) in *N. benthamiana* leaves. Results are shown as means ± SE (*n* = 6). “**” indicates a significant difference (*p* < 0.01, Student’s *t*-test) between HaWRI1 and control (reporter without addition of HaWRI1). (**E**) Binding of the HaWRI1 AP2 domain (amino acids 52–234) to probe containing AW-box in *proBCCP2* using EMSA. (**F**) Schematic diagram of HaWRI1. Acidic amino acid residues in the C-terminal HaWRI1 are highlighted in red color. (**G**) Transactivation assay of HaWRI1 in yeast cells. HaWRI1 full-length and a series of HaWRI1 truncated variants were subcloned to vector fused with yeast GAL4 DNA binding domain (BD). Transactivation activity was measured via β–galactosidase assay of liquid cultures. Results are shown as means ± SE (*n* = 3).

Transient production of plant oil regulators in *N. benthamiana* leaves, followed by lipid analysis, is an efficient and vigorous method to investigate the function of fatty acid biosynthetic regulators [34,35,36,38,40]. We hence investigated the HaWRI1 function in mediating oil accumulation via the established *N. benthamiana* transient expression platform [34,35,38]. We examined oil droplet formation in *N. benthamiana* leaves after agroinfiltration of the vector overexpressing *HaWRI1*. As shown by BODIPY staining, only a few oil droplets were detected in the control leaves infiltrated with the empty vector (EV). By comparison, a significantly elevated number of oil droplets were visible in leaves transiently expressing *HaWRI1* (Figure 3A). We next conducted a quantitative analysis of the TAG accumulation in *N. benthamiana* leaves after agroinfiltration. As shown in Figure 3B, transient expression of *HaWRI1* stimulated oil production in *N. benthamiana* leaves, which was consistent with the result of BODIPY staining (Figure 3A).

### 2.5. HaWRI1 Binds to and Activates proBCCP2 and proPKP-β1

We also examined the transactivation activity of HaWRI1 using a *N. benthamiana* transient expression system, in which the *CaMV* 35S promoter-driven *HaWRI1* (*35S:HaWRI1*) was co-expressed with the reporters, of which the *LUC* gene expression is driven by the promoters of WRI1 target genes (*proBCCP2* and *proPKP-β1*). Our results indicated that co-expression of *HaWRI1* with *proBCCP2:LUC* or and *proPKP-β1:LUC* resulted in a substantial elevation in LUC activity, suggesting that HaWRI1 transactivated both *proBCCP2* and *proPKP-β1* in plant cells (Figure 3D). We next investigated the binding capacity of HaWRI1 to *proBCCP2* using EMSA, in which a biotin-labeled probe containing the AW-box of *proBCCP2* was incubated with purified recombinant His-HaWRI1^52−234^, and the protein–DNA complex was visualized after electrophoresis. His-HaWRI1^52−234^ bound to the probe in EMSA (Figure 3E). With a gradual increase of the unlabeled probe (10× to 60× of the cold probe), we observed the demolishing of the labeling signal, suggesting a high binding specificity of His-HaWRI1^52−234^ to the labeled probe (Figure 3E).

### 2.6. TAD Is Located at the C-terminus of HaWRI1

We analyzed the functional domains vital for the transactivation activity of HaWRI1. As shown in Figure 3F, the C-terminal region of HaWRI1 is enriched in acidic amino acid residues (D or E), a common feature of TADs [41]. We next used a GAL4-based one-hybrid system to determine the TAD of HaWRI1. The initial attempt of deletion of residues 311–391 (HaWRI1^1−310^) led to the abolishment of transactivation activity, and the HaWRI1^311−391^ truncated variant retained strong transactivation activity (Figure 3G). These data suggested the importance of C-terminal HaWRI1 (HaWRI1^311−391^) in transactivation. In summary, our evidence suggested that HaWRI1^311−391^ was indispensable for conferring transactivation activity and hence designated as TAD of HaWRI1.

### 2.7. Overexpression of HaWRI1 in Arabidopsis Leads to Enhanced Seed Oil Accumulation

**Figure 4:**
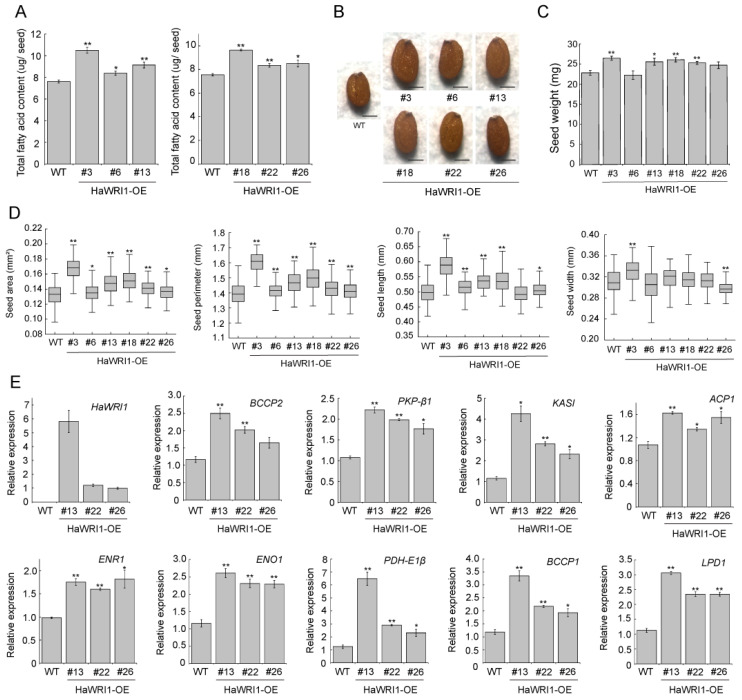
Functional analysis of transgenic Arabidopsis overexpressing *HaWRI1* (HaWRI1-OE). (**A**) Fatty acid content of seeds of Arabidopsis HaWRI1-OE lines. Results are shown as means ± SE (*n* = 3–4). (**B**) Mature dry seeds of WT and HaWRI1-OE lines. Scale bar is 200 μM. (**C**) Quantitative analysis of seed mass WT and HaWRI1-OE lines. Seed mass was attained by measuring 100 seeds per sample, and results are shown as means ± SE (*n* = 4–5). (**D**) Quantitative analysis of seed size of WT and HaWRI1-OE lines. Seed size is denoted by seed area, perimeter, length, and width with box plots displaying medians (lines), interquartile ranges (boxes) and 1.5× interquartile ranges (whiskers) of WT and HaWRI1-OE lines (*n* = 100). “*” and “**” indicate significant differences (*p* < 0.05 and *p* < 0.01, respectively, Student’s *t*-test) between WT and HaWRI1-OE lines. (**E**) Pooled siliques (10 DAF) from WT and HaWRI1-OE lines were used for the assay. Transcript levels of various WRI1 target genes (*BCCP2*, *PKP-β1*, *ACP1*, *KASI*, *ENR1*, *ENO1*, *PDH-E1β*, *LPD1*, *BCCP1*) were measured by qRT-PCR. Results are shown as means ± SE (*n* = 3). “*” and “**” indicate significant differences (*p* < 0.05 and *p* < 0.01, respectively, Student’s *t*-test) between WT and HaWRI1-OE lines in terms of expression of WRI1 target genes.

To further corroborate the function of HaWRI1 *in planta*, we generated stable transgenic Arabidopsis plants overexpressing *HaWRI1* under the control of the seed-specific glycinin promoter [42]. We successfully obtained multiple independent *HaWRI1* overexpression lines (HaWRI1-OE) and subsequently measured the fatty acid content in their seeds. As shown in Figure 4A, the seed fatty acid content in three HaWRI1-OE lines were significantly higher than that in wild-type (WT). As measured by the microscope, HaWRI1-OE lines produced larger seeds (Figure 4B). Seed weight measurement showed that multiple HaWRI1-OE lines displayed significantly higher seed weight than that of WT (Figure 4C). In addition, we performed a detailed quantitative analysis of several seed parameters of the HaWRI1-OE lines. The majority of HaWRI1-OE lines exhibited increased area, perimeter, length, and width compared to WT (Figure 4D).

We conducted qRT-PCR to examine the expression of the known WRI1 targets in developing seeds of HaWRI1-OE lines. Expression of numerous WRI1 target genes (including *BCCP2*, *ACP1*, *PKP-β1*, *KASI*, *ENR1*, *ENO1*, *PDH-E1β*, *LPD1*, *BCCP1*) exhibited significant upregulation in HaWRI1-OE compared to WT (Figure 4E). On the other hand, the expression of *FAD3* and *DGAT1*, two genes known to be not WRI1 targets, remained unchanged (Supplementary Figure S6). Together, our evidence from stable transgenic plants suggested that HaWRI1, in a seed-specific expression manner, was capable of boosting seed oil production by activating the expression of genes involved in fatty acid biosynthesis.

## 3. Discussion

Although the fatty acid accumulation pattern during sunflower seed development is well documented [7,8,43,44], the molecular mechanism of gene regulation underlying sunflower seed oil accumulation remains to be fully investigated. Seed oil content and quality improvement have been major goals of sunflower breeding, necessitating the molecular characterization of the key regulators governing seed oil accumulation in sunflowers. Here, we characterized the sunflower HaWRI1, with only 52% sequence identity to AtWRI1 protein. In silico analysis also revealed that protein structural hallmarks and functional motifs/domains (e.g., IDRs and “VYL”) were conserved in HaWRI1, despite divergence of AtWRI1 and HaWRI1 at the C-terminus (Supplementary Figures S1 and S2). RNA-seq and qRT-PCR analyses showed that *HaWRI1* exhibited a tissue-specific expression that is the highest in developing seeds. The expression pattern of *HaWRI1* was correlated with WRI1 target genes involved in fatty acid biosynthesis (Figure 2), suggesting that HaWRI1 plays a key role in regulating seed oil accumulation in sunflower. The expression of *AtWRI1* has been shown to be regulated by several seed maturation master regulators, such as LEC1, LEC2, and FUS3 in Arabidopsis seeds [16,45,46,47,48,49]. However, in contrast to Arabidopsis, we did not identify a sunflower ortholog of LEC2 in our analysis; hence, it is unclear whether a LEC2 ortholog exists in sunflower to regulate fatty acid biosynthesis and seed development. Nonetheless, we found that *HaLEC1* and *HaFUS3* displayed high expression levels as *HaWRI1* in developing sunflower seeds (Supplementary Figure S7), suggesting that *HaWRI1* is transcriptionally controlled by these upstream transcriptional regulators.

The present study also revealed that HaWRI1 transcriptionally controls plant oil biosynthesis. The transient overproduction of AtWRI1 and some WRI1 orthologs lead to TAG accumulation in *N. benthamiana* leaves [21,24,34,40]. Here, our results showed that ectopic expression of *HaWRI1* significantly stimulated the TAG accumulation in leaves of *N. benthamiana* (Figure 3A,B). Our molecular evidence also indicated that HaWRI1 effectively transactivated the promoters of WRI1 targets genes in plant cells (Figure 3D). The DNA binding specificity and affinity of WRI1 are of great importance for its function. Biochemical assay indicated that the AP2 domain of HaWRI1 was able to bind to the promoters of HaWRI1 target genes containing AW-box (Figure 3E). Further functional assay characterized a C-terminal TAD in HaWRI1 (Figure 3F,G). Moreover, our evidence indicated that seed-specific expression of *HaWRI1* led to increased seed oil accumulation in stable transgenic Arabidopsis, as well as the upregulation of various genes involved in fatty acid biosynthesis (Figure 4A,E), suggesting a possible role of HaWRI1 in oil bioengineering. Markedly, multiple HaWRI1-OE lines also exhibited increased seed mass and size (Figure 4B–D). This finding was similar to discoveries reported previously in transgenic Arabidopsis or other plant species overproducing WRI1s, possibly due to increased oil accumulation in seeds [20,22,23,50,51].

The AW-box, which is vital for WRI1 transactivation, is generally located close to the transcriptional start sites (TSS) of diverse WRI1 target genes [17]. Here, we surveyed the promoter region of several HaWRI1 targets and found that the promoters of HaWRI1 targets contain at least one AW-box that is adjacent to TSS (Supplementary Figure S8). Thus, our findings suggested that AW-boxes are conserved among HaWRI1 target genes and indispensable for fatty acid biosynthesis.

In conclusion, our work provided genetic and biochemical evidence to verify that HaWRI1 has a conserved function in transcriptional control of TAG accumulation *in planta*. TAG has great importance for human diets as well as other substantial uses such as energy generation. Given the importance of sunflower in vegetable oil production, the development of sunflower varieties with high seed oil content has been an important breeding task for decades. Compared to a single-enzyme strategy, manipulation of transcription regulators has been considered to offer a desirable resolution for plant oil yield improvement [31,52,53]. In this work, we used a seed-specific promoter to drive *HaWRI1* expression, which resulted in increased seed oil accumulation. As such, the work presented in this study provided a promising candidate, *HaWRI1*, for sunflower oil bioengineering in the future.

## 4. Materials and Methods

### 4.1. Plant Materials

Arabidopsis (Columbia ecotype) and *Nicotiana benthamiana* plants were grown in a growth chamber at 23 °C with a photoperiod of 16 h light (100–150 μmol m^−2^ s^−1^ illumination)/8 h dark. Seed sterilization, transformation, and germination were carried out using the methods described previously [26]. Transformed Arabidopsis seeds were screened by their red fluorescence under green light via a portable Dual Fluorescent Protein Flashlight (Nightsea, Lexington, MA, USA). Sunflower (*Helianthus annuus*) plants (Boutique Garden, Kwun Tong, Hong Kong SAR, China) were grown on potting mix under natural conditions.

### 4.2. Bioinformatic Analysis

Intrinsically disordered region (IDR) analysis was performed using PONDR-VL3 predictor [54]. MEGA X software [55] was used for the construction of a phylogenetic tree through the neighbor-joining method with bootstrap values set at 1000 replicates. RNA-seq datasets of five different tissues (seed, root, leaf, stamen, and pistil) were retrieved from the NCBI sequence read archive (SRA) database (accession number PRJNA483306). Heatmap analysis of *HaWRI1* and oil biosynthesis-related gene expression in different tissues was carried out using pheatmap package with Euclidean distance and complete linkage as distance measure and clustering methods [56].

### 4.3. Plasmid Construction

The coding sequence (CDS) of *HaWRI1* was synthesized to pTwist ENTR by Twist Bioscience to obtain an entry construct. The *HaWRI1* entry construct was introduced to pEarleyGate binary vectors (pEarleyGate100 and pEarleyGate104) [57]. *HaWRI1* was also amplified by PCR and subcloned to the binary vector pBinGlyRed1 for the *HaWRI1* expression driven by an embryo-specific glycinin promoter [42]. Truncated *HaWRI1*^52−234^ was subcloned into the pET41a-6×His vector [58] to generate His-tagged recombinant protein in *E. coli*. To generate reporter constructs using the promoters of *biotin carboxyl carrier protein isoform 2 (proBCCP2*) and *plastidic pyruvate kinase beta subunit 1* (*proPKP-β1*), PCR amplified 2 kb promoters were subcloned into the pGreenII 0800-LUC vector [59]. Supplementary Table S1 provides a list of primers used for plasmid construction in this study.

### 4.4. Transient Expression in N. benthamiana and Confocal Microscopy

*Agrobacterium tumefaciens*-mediated transient expression in *N. benthamiana* leaves and confocal microscopy experiments were performed as previously described [38].

### 4.5. BODIPY Staining

*N. benthamiana* plants were put in a plant growth chamber after the agroinfiltration. Leaf discs were collected 4 days post infiltration, and *Agrobacterium*-infiltrated leaf discs were stained with 5 µg/µL BODIPY 493/503 (Sigma-Aldrich, Missouri, MO, USA) for 5 min in the dark by vacuum infiltration. The stained leaf discs were washed with water three times, prior to the lipid droplet observation under a confocal microscope.

### 4.6. Yeast Transactivation Assay

The HaWRI1 variants were subcloned into pGBKT7 vector. The transactivation activities of the HaWRI1 variants were measured through the liquid culture assay as previously described [34].

### 4.7. Recombinant Protein Production and EMSA

His-HaWRI1^52−234^ recombinant protein was produced in *E coli* [BL21 (DE3)], extracted, and purified as previously described [34,58]. The 5′end biotin-labeled (hot) and unlabeled (cold) probes of the *proBCCP2* AW-box (5′-TACTTCCTCGGTTTCATCGTCCAC-3′) were used for EMSA. The standard binding reaction (20 µL) contained 0.05 µg/µL poly(dI-dC), 15 mM HEPES-KOH (pH 7.5), 7.5 mM KCl, 0.5 mM EDTA, 5% glycerol, 2 mM dithiothreitol, 1 µg/µL BSA, 2 fmol/µL of the hot DNA probe and 280 ng of His-HaWRI1^52−234^. The reaction mixture was incubated on ice for 30 min. The DNA–protein complexes were resolved on 5% (*w*/*v*) non-denaturing polyacrylamide gels and subsequently transferred to nylon membranes. The band shifts were detected by a chemiluminescent nucleic acid detection module (Thermo Scientific, Roskilde, Denmark).

### 4.8. Measurement of Seed Weight and Size

Arabidopsis seed weight and size were measured following the methods described previously [60] with slight modifications. In brief, one hundred dry seeds were used to measure the average seed weight by a GR-200 Analytical Balance (A&D, Tokyo, Japan). Regarding seed size determination, approximately one hundred seeds were spread on a glass slide and scanned (together with a scale bar) by an Epson Perfection V600 Photo Scanner. The seed length, width, perimeter, and area were analyzed using ImageJ software (http://imagej.nih.gov/ij/, last accessed on 15 January 2022).

### 4.9. Gene Expression Analysis

For spatial gene expression analyses, developing *H. annuus* seeds at 18 DAF and 3.5-week-old plants were used to obtain seed, leaf, root, and stem materials. Ray florets and disk florets were harvested from 8 to 8.5-week-old *H. annuus* plants. Plant samples were harvested and frozen in liquid nitrogen and stored at −80 °C until use for RNA extraction. Samples were ground in liquid nitrogen to fine powder, and total RNA was extracted using a Monarch Total RNA Miniprep Kit (New England Biolabs, Ipswich, MA, USA) according to the manufacturer’s instructions. Genomic DNA contamination was removed using the DNase I (New England Biolabs, Ipswich, MA, USA) following the manufacturer’s instructions. First-strand cDNA was synthesized using the qScript cDNA Synthesis Kit (Quantabio, Beverly, MA, USA). Subsequent quantitative real-time PCR (qRT-PCR) was performed using the Luna Universal qPCR Master Mix (New England Biolabs, Ipswich, MA, USA) following the manufacturer’s instructions. *AtPP2A* and *HaActin7* were used as internal controls to normalize the gene expression in Arabidopsis and sunflower, respectively. The primers used for qRT-PCR are provided in Supplemental Table S2.

### 4.10. Dual-LUC Assay

Transient dual-luciferase assay in *N. benthamiana* leaves was performed following the protocols described previously [61,62] with slight modifications. *N. benthamiana* plants were put in a plant growth chamber after the agroinfiltration. Leaf discs were collected 3 d post infiltration for the dual-LUC experiment using the Dual-Luciferase Reporter 1000 Assay System (Promega, Madison, WI, USA). Briefly, three leaf discs (5–6 mm in diameter) from the agroinfiltration areas were removed and then ground to fine powder in liquid nitrogen. The powder was subsequently homogenized in 100 µL Passive Lysis buffer (Promega, Madison, WI, USA). Next, 5 µL of the extract was mixed with 40 µL of Luciferase Assay Buffer, and the firefly LUC activity was quantified by a cell imaging multimode plate reader (BioTek Cytation 5, Santa Clara, CA, USA). The reaction was stopped by adding 40 µL Stop and Glo Buffer (Promega, Madison, WI, USA), and the Renilla (REN) LUC activity was quantified. The firefly LUC activity was normalized to the REN LUC activity.

### 4.11. Fatty Acid Analysis

Fatty acid analysis of Arabidopsis seeds or *N. benthamiana* leaves was performed as previously described [26,34].

### 4.12. Accession Numbers

Sequence information are identified in Arabidopsis Information Resource database (www.arabidopsis.org, last accessed on 15 January 2022), GenBank/EMBL databases or Phytozome (https://phytozome-next.jgi.doe.gov/, last accessed on 15 January 2022) under accession numbers: AtWRI1 (At3G54320); BdWRI1 (Bradi4g43877); BnWRI1 (ABD16282.1); CnWRI1 (JQ040545); CsWRI1a (KY129795); CsWRI1b (KY129796); CsWRI1c (KY129797); GmWRI1a (Glyma08g227700); GmWRI1b (Glyma15g221600); HaWRI1 (HanXRQChr14g0446701); JcWRI1 (AIA57945.1); VvWRI1 (CBI32013.3); ZmWRI1a (GRMZM2G124524); ZmWRI1b (GRMZM2G174834); HaBCCP2 (HanXRQChr02g0042491); HaACP1 (HanXRQChr14g0445471); HaKASI (HanXRQChr17g0564321); HaPKP-β1 (HanXRQChr13g0393011); HaActin7 (HannXRQChr14g0446641); AtBCCP2 (AT5G15530); AtPKP-β1 (AT5G52920); AtACP1 (AT3G05020); AtKASI (AT5G46290); AtENR1 (AT2G05990); AtENO1 (AT1G74030); AtPDH-E1β (AT1G30120); AtBCCP1 (AT5G16390); AtLPD1 (AT3G16950); AtFAD3 (AT2G29980); AtDGAT1 (AT2G19450); AtPP2A (AT1G13320).

## Data Availability

All data supporting the findings of this study are available within the paper and within its supplementary material published online.

## Supplementary Materials

The following are available online at https://www.mdpi.com/article/10.3390/ijms23063054/s1.
