# Supplementary material for: Sunflower WRINKLED1 Plays a Key Role in Transcriptional Regulation of Oil Biosynthesis
Source: Int J Mol Sci. 2022 Mar 11;23(6):3054. doi: 10.3390/ijms23063054 (PMC8951541; doi:10.3390/ijms23063054)
Supplement: Supplementary file 1 [file ijms-23-03054-s001.zip › ijms-1606041-supplementary.pdf]

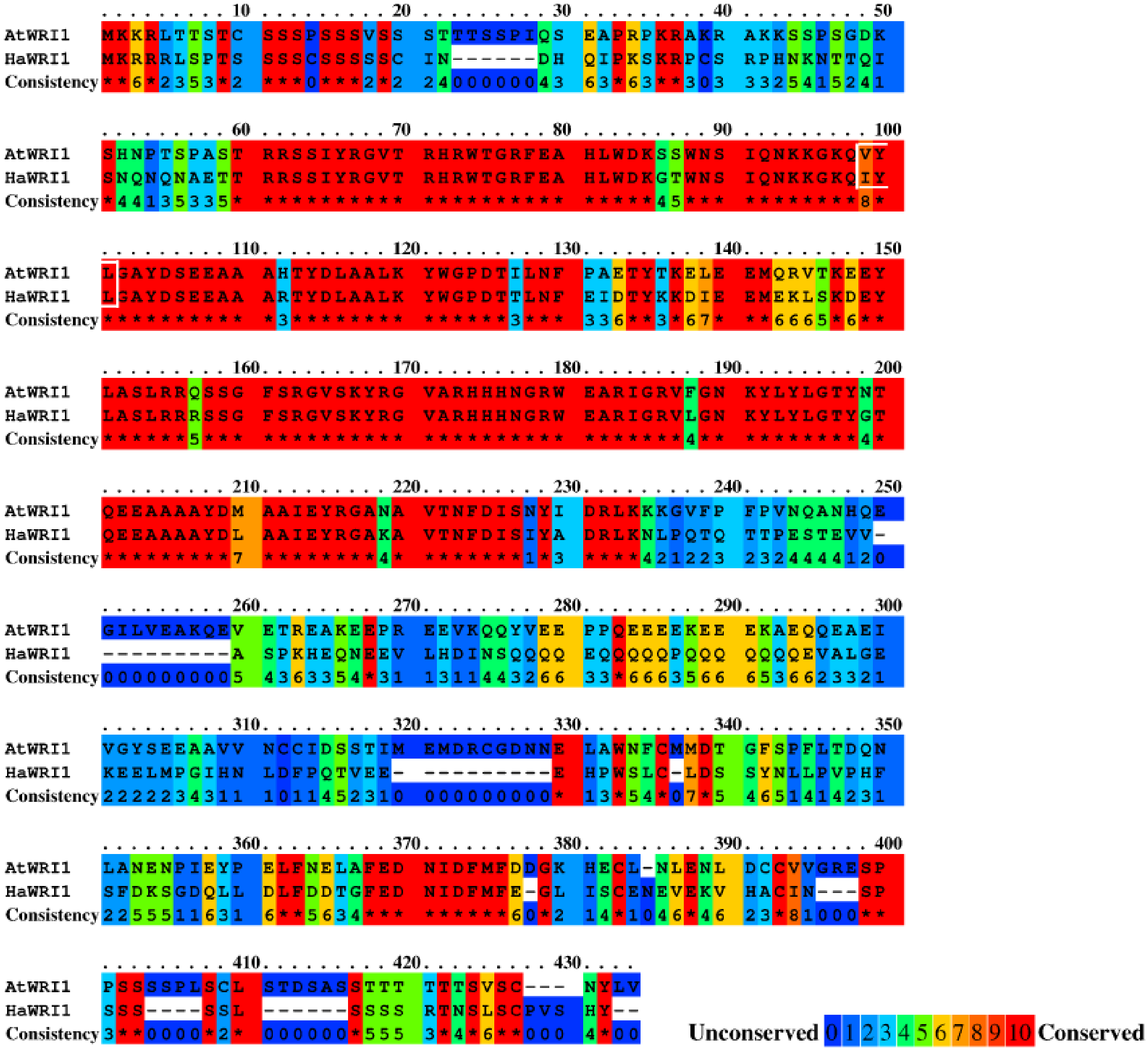

Figure S1. Alignment of protein sequences of AtWRI1 and HaWRI1. A) Protein alignment was conducted by the PRALINE program. Conservation of residues is designated by various colors as demonstrated by the scale bar. Essential functional motif "VYL" in AtWRI1 is conserved in HaWRI1 as "IYL".

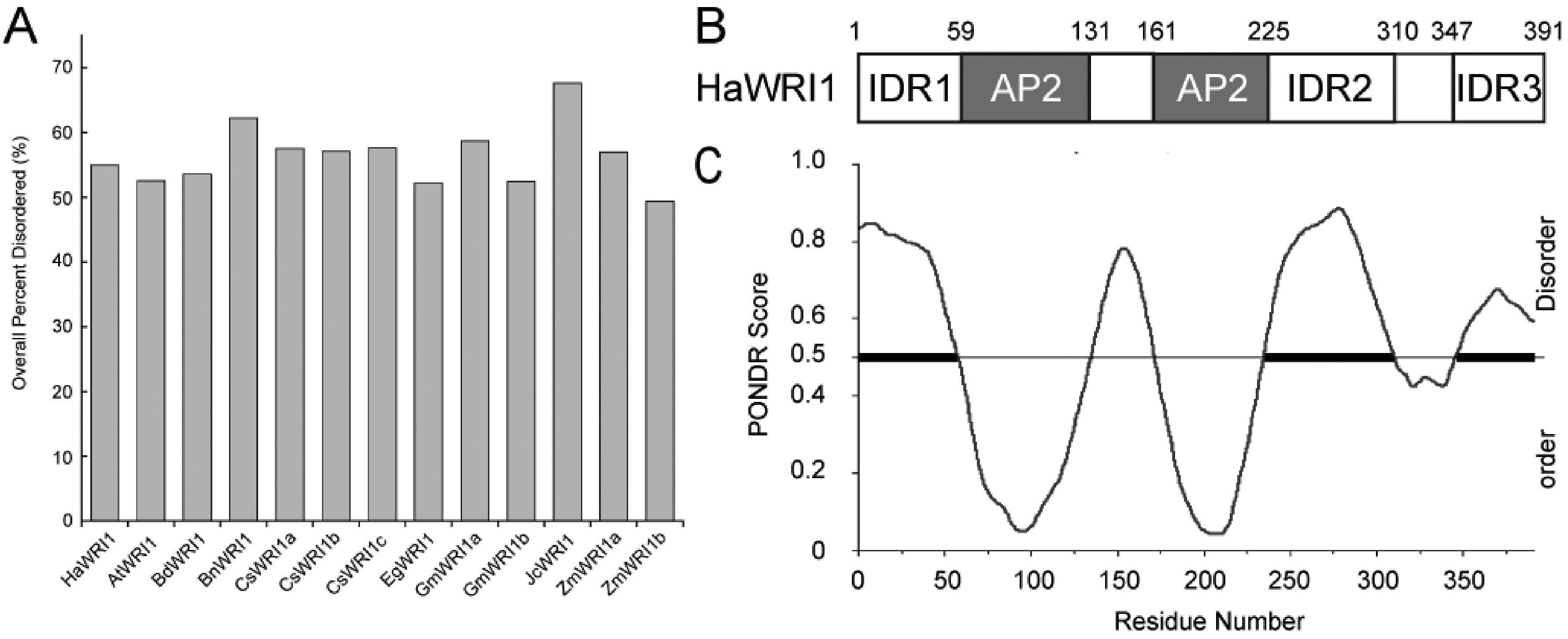

Figure S2. Intrinsic disorder analysis of HaWRI1. A) Overall disorder prediction by PONDR-VL3 program. B) Schematic diagram of HaWRI1. C) Disorder of HaWRI1 predicted by PONDR-VL3 program.

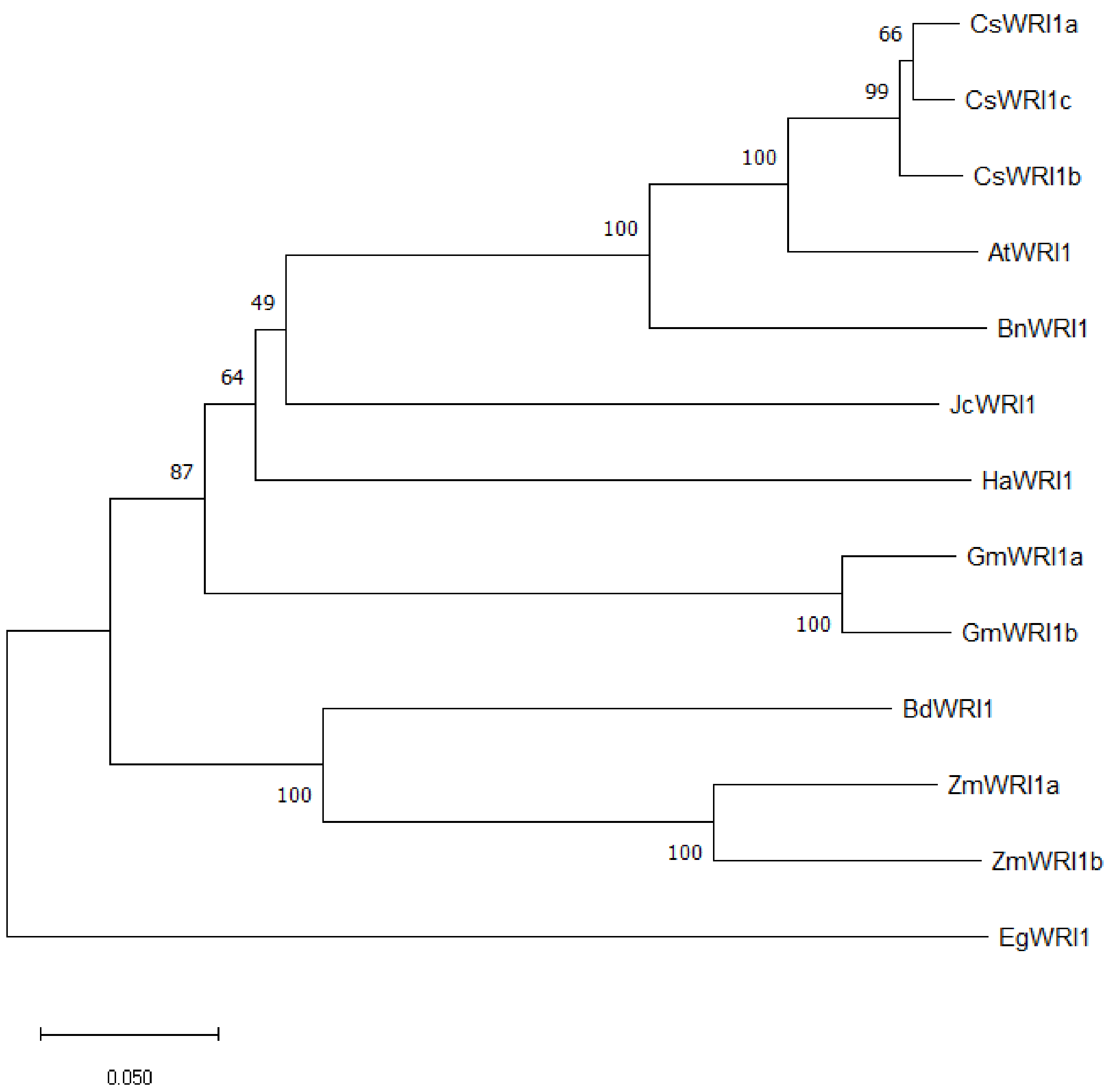

Figure S3. Phylogenetic analysis of WRI1s identified from various plant species. The phylogenetic tree was generated using various WRI1 protein sequences. The tree was built by MEGA X using the neighbor-joining method. The number of bootstrap replicates is 1000.

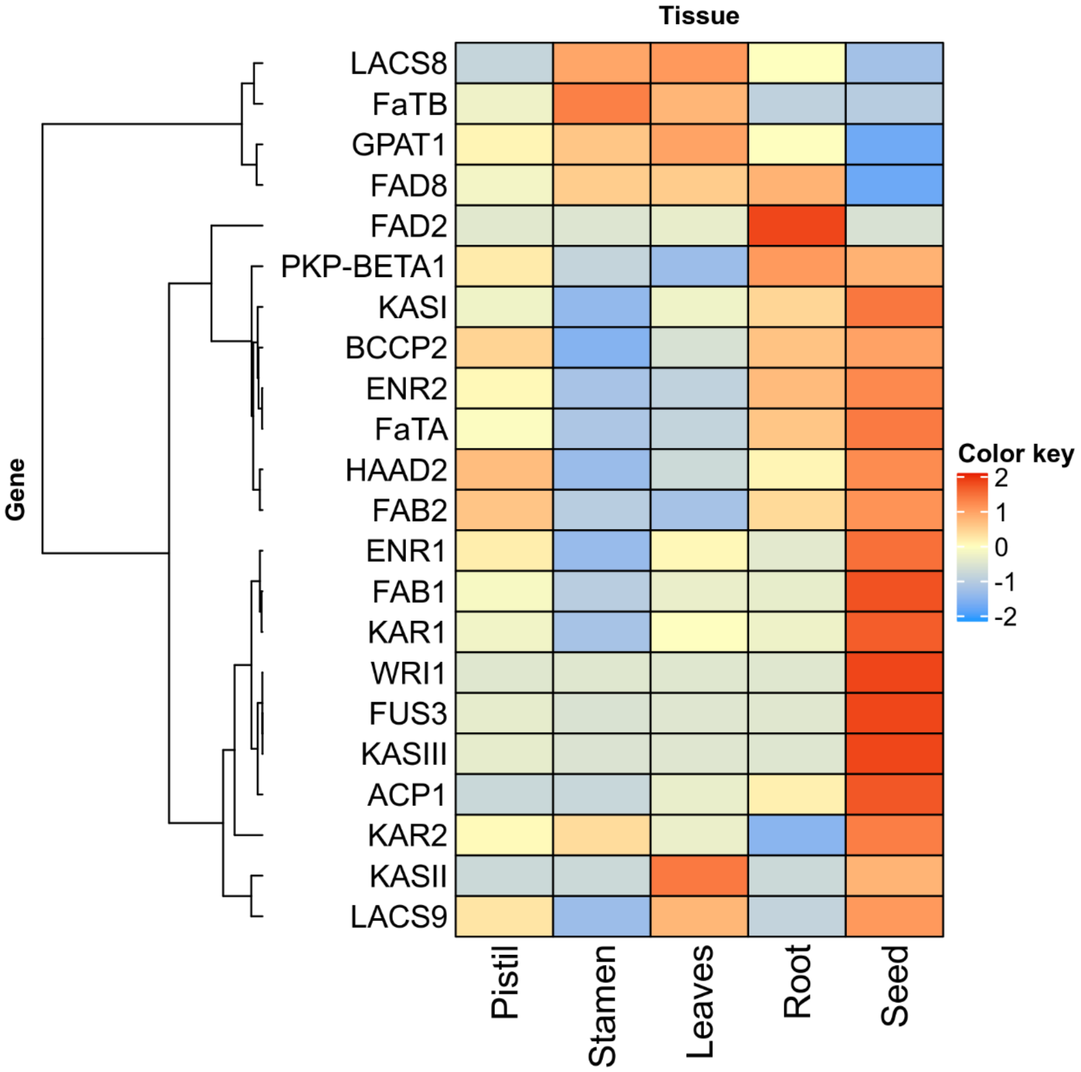

Figure S4. Expression analysis of *WRI1* and other oil biosynthesis-related genes in different sunflower tissues. Gene Expression heatmap of 22 oil biosynthesis-related genes in 5 tissues are shown. Y-axis represents the genes while X-axis-tissue shows the tissue samples. Orange represents higher expression, and blue represents lower expression. The RNA-seq datasets deposited in the sequence read archive database (accession number PRJNA483306) were analyzed to get the expression value.

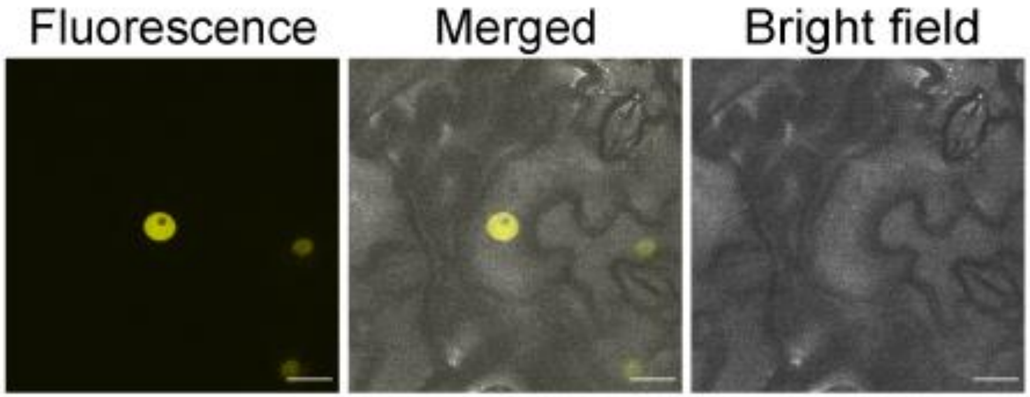

Figure S5. Subcellular localization of YFP-HaWRI1 via *N. benthamiana* transient expression experiment. Confocal images are from *N. benthamiana* epidermal cells transiently producing YFP-HaWRI1. Scale bar is 20 μM.

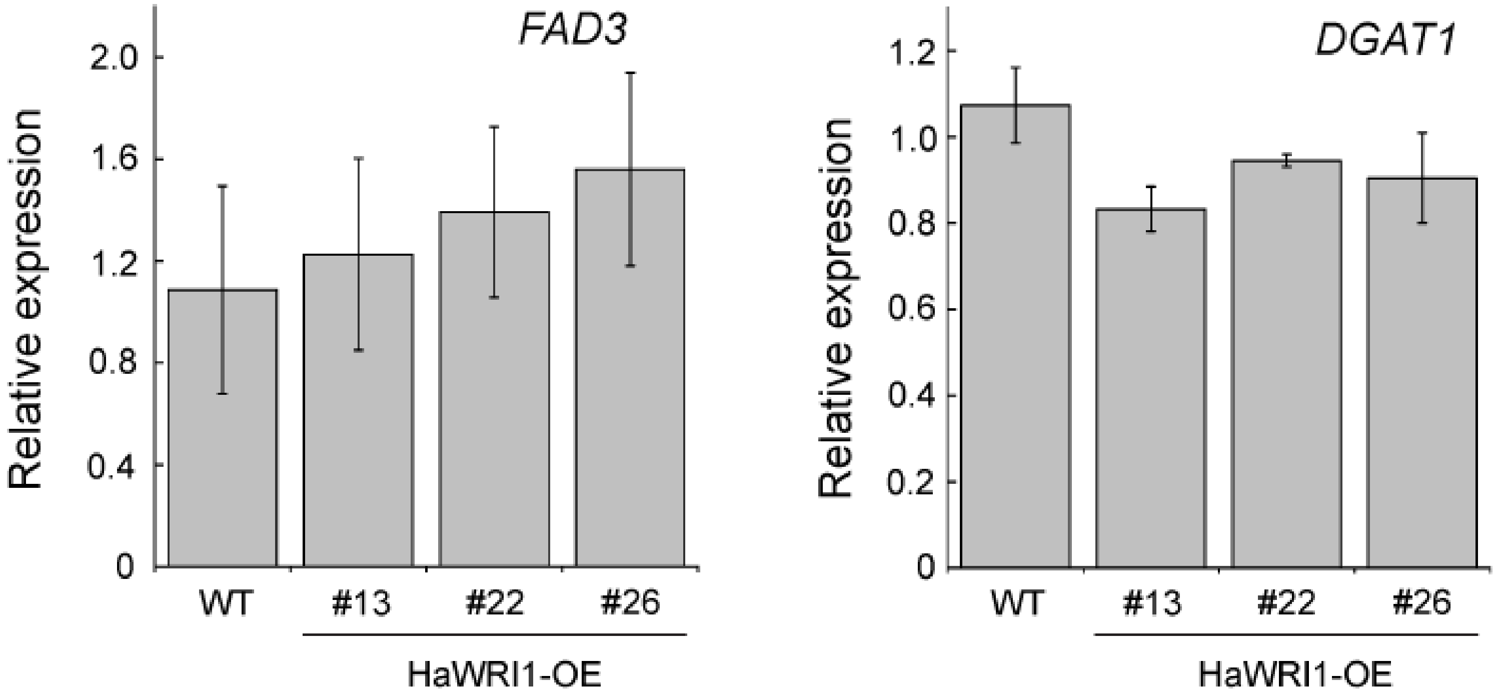

Figure S6. Expression analysis of non-WRI1 targets in wild-type (WT) and transgenic Arabidopsis overexpressing *HaWRI1* (HaWRI1-OE). Pooled siliques (10 DAF) from WT and HaWRI1-OE lines were used for the assay. Expression level of non-WRI1 targets (*FAD3 and DGAT1*) was quantified by quantitative real-time PCR (qRT-PCR). Results are shown as means ± SE (n=3).

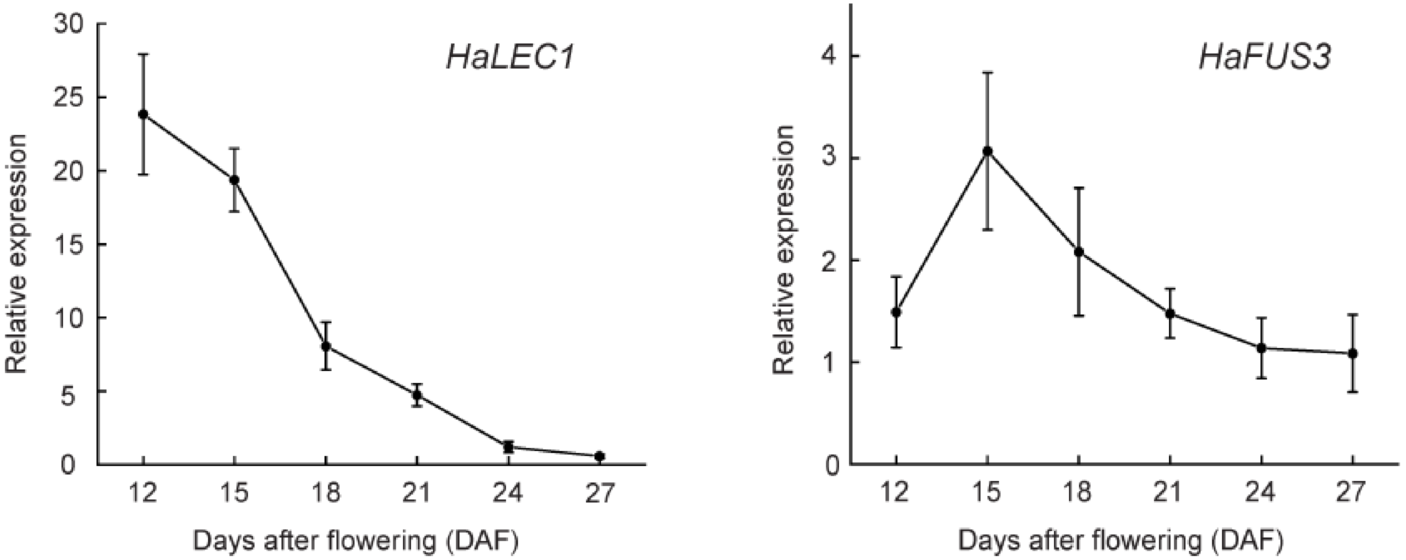

Figure S7. Expression analysis of *LEC1* and *FUS3* in developing seeds of *H. annuus*. Expression level of *HaLEC1*, and *HaFUS3* at various stages during seed development [12-27 days after flowering (DAF) as indicated] was quantified by qRT-PCR. Results are shown as means ± SE (n=4-5).

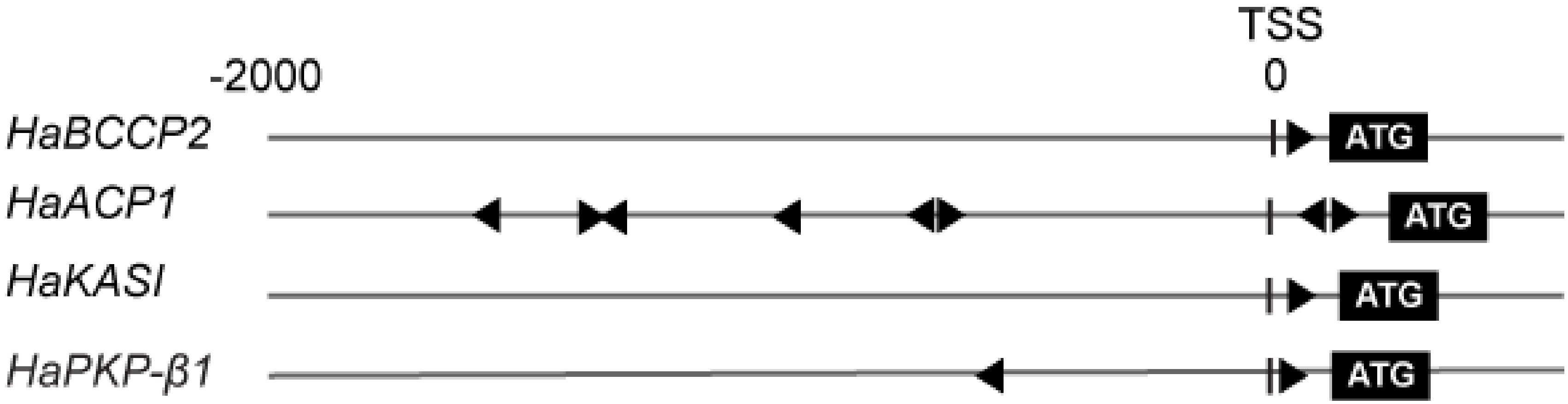

Figure S8. *In silico* analysis of the AW-box in the promoters of HaWRI1 target genes. The region from 2 kb upstream of the TSS (transcription start site) was examined for each target gene. Schematic diagram illustrates 2kb promoter region of HaWRI1 targets. Positions of AW-box are indicated by arrowheads. AW-boxes in the sense strand and antisense strand are indicated by arrowheads pointing to the right and left, respectively.

Table S1. Primers used for plasmid construction in this study.

Primer Name	Sequence 5' to 3'
HaWRI1-FW	5'-CGCGAATTCATGAAAAGAAGAAGGTTG-3'
HaWRI1-RV	5'-CGCCTCGAGTCAATAATGTGAAACCGG-3'
HaWRI1$^{52-234}$-FW	5'-ATTGGATCCGAAACTACACGCAGGAGCTCT-3'
HaWRI1$^{52-234}$-RV	5'-ATTCTCGAGCTATTGGGTTTGTGGCAAGTT-3'
HaWRI1-FW (yeast assay)	5'-GCCGAATTCATGAAAAGAAGAAGG-3'
HaWRI1-RV (yeast assay)	5'-ATTGGATCCTCAATAATGTGAAAC-3'
HaWRI1$^{1-310}$-RV	5'-ATTGGATCCTCAACACAAGCTCCA-3'
HaWRI1$^{311-391}$-FW	5'-GCCGAATTCTTGGATTCAAGTTAT-3'
proAtBCCP2-FW	5'-ATTCTCGAGGAATATGGCGAGCAATA-3'
proAtBCCP2-RV	5'-ATTGGATCCTGTTGAGACAGTGGACG-3'
proAtPKP-β1-FW	5'-ATTCTCGAGTCAGCATCAATACCAAC-3'
proAtPKP-β1-RV	5'-ATTGGATCCTTCTGATTTTGAAGAGA-3'

Table S2. Primers used for quantitative real-time PCR (qRT-PCR) in this study.

**Primer name**	**Sequence 5' to 3'**
HaWRI1-FW	5'-TTACAGGGGTGTCACAAGGC-3'
HaWRI1-RV	5'-CAGCAGCCTCTTCGCTATCA-3'
HaLEC1-FW	5'-GCAACAGCCGGATATGAAGC-3'
HaLEC1-RV	5'-TGCTATCGGCATAAAGCGGT-3'
HaFUS3-FW	5'-GGTGTAAGTGTGCTTGGTTCG-3'
HaFUS3-RV	5'-TTTAGCACACCCACATCGCT-3'
HaBCCP2-FW	5'-CCTTTGCTGTCTTCTGCACG-3'
HaBCCP2-RV	5'-GCGGACACTTTCATAACGCC-3'
HaACP1-FW	5'-TTCACCGGATCATCCACCCTA-3'
HaACP1-RV	5'-CTGTCTCTGGTTTGGCAGCA-3'
HaKASI-FW	5'-TGTGCTTGTTGGGACAGGAA-3'
HaKASI-RV	5'-ATCAATAGCGAGCAGAGCCG-3'
HaPKP-β1-FW	5'-TCAAGGCCCTTAGTGAAGCG-3'
HaPKP-β1-RV	5'-GGTCTCCGTGGGACATGTTT-3'
HaActin7-FW	5'-AGCTCGAGACTGCAAAGAGC-3'
HaActin7-RV	5'-ACGGAATCTTTCGGCACCAA-3'
AtBCCP2-FW1	5'-GCAGCTCGACTGTGAGATCG-3'
AtBCCP2-RV1	5'-GTCTGCCATTACAGGAGGCAT-3'
AtPKP-β1-FW	5'-ACTGTGAGATCCGCTCGTGTTG-3'
AtPKP-β1-RV	5'-AACCTCTCAAGCTGCTCCTCTCTG-3'
AtACP1-FW1	5'-GAAGCAACTATCACTTACACC-3'
AtACP1-RV1	5'-GAATGGATACCGTGTCGAGAG-3'
AtKASI-FW	5'-TGAAGGAGCTGGTGTTCTGGTG-3'
AtKASI-RV	5'-TTGGAGCACCACGTTTCATTGC-3'
AtENR-FW	5'-TCAAGGAAAGGCTATCTCGCTGCT-3'
AtENR-RV	5'-TCACTCTCTAGTGCAGCTTTGGCA-3'
AtFATA-FW	5'-TGTCCTCAAGAACCGAGGTTAGC-3'
AtFATA-RV	5'-ACTGAGCCGGATCTTCGAGTTTC-3'
AtENO1-FW	5'-TTAGTCACAGGAGTGGCGAGAC-3'
AtENO1-RV	5'-TCTTCGATACGGAGAAGCTGGTTG-3'
AtPDH-E1β-FW	5'-TCTGCGAGCACTGGACATGAAC-3'
AtPDH-E1β-RV	5'-TCCATCTCTTCTTCCAGACCTTCC-3'

AtLPD1-FW	5'-TGCACATCCAACACTCTCTGAGG-3'
AtLPD1-RV	5'-TCGTAGCATGACTTTCAACCTTGG-3'
AtBCCP1-FW	5'-ATTGTCGCAGAAGACGGCAAGC-3'
AtBCCP1-RV	5'-ACGGTTGAACCACAAACAGAGGAG-3'
AtFAD3-FW	5'-TTTGTTCTCGGCCACGACTGTG-3'
AtFAD3-RV	5'-TGGTGTGTCCGGTGGCTTATTC-3'
AtDGAT1-FW	5'-CCGACGGTCGATACGTAAACGTAG-3'
AtDGAT1-RV	5'-GCCGGAGATAATAACGGTGG-3'
AtPP2A-FW	5'-TAACGTGGCCAAAATGATGC-3'
AtPP2A-RV	5'-GTTCTCCACAACCGCTTGGT-3'
